# Chaperone-mediated autophagy dysfunction in imiquimod-induced psoriasiform dermatitis

**DOI:** 10.1080/27694127.2025.2544061

**Published:** 2025-08-25

**Authors:** Wei Zhao, Kainan Liao, Wei Song, Jing Wang, Chunlin Cai, Fusheng Zhou, Dandan Zang, Deping Xu, Haisheng Zhou

**Affiliations:** a Department of Biochemistry and Molecular Biology, School of Basic Medical Sciences of Anhui Medical University, Hefei, China; b Department of Pathophysiology, School of Basic Medical Sciences, Anhui Medical University, Hefei, China; c Key Laboratory of Dermatology, Anhui Medical University & Ministry of Education, Hefei, China; d Center for Scientific Research, Anhui Medical University, Hefei, China; e Clinical Laboratory, The second People’s Hospital of Hefei, Hefei Hospital Affiliated to Anhui Medical University, Hefei, China

**Keywords:** Chaperone-mediated autophagy, keratinocyte, NF-κB, psoriasis, toll-like receptor 7

## Abstract

Psoriasis is a chronic inflammatory skin disease characterized by abnormal differentiation and hyperproliferation of epidermal keratinocytes. Autophagy plays a critical role in regulating the functions of immune cells, endothelial cells, and especially keratinocytes, contributing to the pathogenesis of psoriasis. However, the role of chaperone-mediated autophagy (CMA) in psoriatic keratinocytes has not been fully explored. Our study, for the first time, revealed that defective CMA is present in imiquimod (IMQ)-induced psoriasiform lesions. Importantly, activation of CMA significantly attenuated IMQ-induced phenotypes both *in vitro* and *in vivo*, including reduced skin lesion severity, decreased keratinocyte proliferation and differentiation, and lower cytokine secretion. Mechanistically, toll-like receptor 7 (TLR7), containing a specific KFERQ-like motif, is a substrate for CMA-mediated degradation. This process modulates IMQ-TLR7 signal activation in keratinocytes. CMA deficiency in psoriasis leads to increased TLR7 levels, which, in turn, enhances TLR7-NF-κB signaling pathway activation, ultimately contributing to dysregulated keratinocyte proliferation, differentiation, and cytokine secretion. This study provides novel evidence that defective CMA is present in IMQ-induced psoriasiform lesions and that CMA activation can attenuate IMQ-induced phenotypes by modulating TLR7 signaling in keratinocytes. These findings highlight the potential of CMA as a therapeutic target for psoriasis.

## Introduction

Psoriasis is a chronic inflammatory skin disease characterized by interleukin (IL)-17-dominant abnormal innate and acquired immunity. This leads to hyperproliferation and aberrant differentiation of epidermal keratinocytes, which contribute to skin inflammation with increased secretion of pro-inflammatory cytokines, such as tumor necrosis factor-α (TNF-α), IL-6, and IL-23^[1–3]^. Keratinocytes, the primary cell population in the epidermis, play a central role in psoriasis^[4–6]^, contributing to the thickening of the epidermis, the formation of scales, and the inflammatory responses observed in lesions. Autophagy, a catabolic process involving the lysosomal degradation of cytoplasmic proteins and organelles, is essential for maintaining cellular and tissue homeostasis^[7]^. It also enables cells to respond to stresses such as hunger and inflammation^[8,9]^. Autophagy regulates the functions of various cells, including mesenchymal stem cells, keratinocytes, T cells, and endothelial cells, and is implicated in the pathogenesis of psoriasis. Mammalian cells utilize three forms of autophagy: macroautophagy (referred to simply as autophagy), microautophagy, and chaperone-mediated autophagy (CMA)^[10]^. Of these, macroautophagy, the best-understood form of autophagy, plays a significant role in the pathogenesis of psoriasis^[11–13]^. Studies have shown that dysfunctional autophagy directly impacts several key processes in psoriasis, including proliferation^[13]^, differentiation^[14–18]^, and inflammatory responses of keratinocytes^[13,19–21]^.

CMA, a specialized form of autophagy, differs significantly from macroautophagy in its selectivity. It specifically targets and degrades client proteins bearing specific CMA motifs, known as pentapeptide motifs, collectively referred to as the KFERQ motif^[22–24]^. CMA substrates are recognized and targeted by the cytosolic chaperone protein HSC70 (heat shock cognate 71 kDa protein), forming an HSC70-substrate complex. This complex then binds to the lysosomal protein LAMP2A (lysosome-associated membrane protein type 2A), facilitating subsequent lysosomal uptake and degradation^[22–25]^. Compared to the well-studied process of macroautophagy, which has been shown to play a protective role against psoriasis by regulating keratinocyte proliferation, differentiation, and cytokine secretion, the role of CMA in psoriasis remains largely unexplored.

Imiquimod (IMQ) is a topical immune response modifier commonly used to induce psoriasiform lesions in mouse models. Mechanistically, IMQ activates Toll-Like Receptor 7 (TLR7), triggering a cascade of events that includes immune cell activation, cytokine release, and keratinocyte hyperproliferation and aberrant differentiation, ultimately leading to the development of psoriasiform lesions^[26–29]^. Additionally, the TLR7-NF-κB pathways are activated in both IMQ-induced psoriasis-like skin inflammation in mice and in normal human epidermal keratinocytes treated with IMQ^[30,31]^. Given the presence of a potential KFERQ-like motif within the human TLR7 protein and the function of CMA in protein degradation, we hypothesize that CMA may regulate TLR7 activation by influencing its degradation. Our current study investigates the alterations in CMA levels within IMQ-induced psoriasiform lesions and the keratinocytes that contribute to this process. We aim to explore the role of CMA in the development of psoriasis, specifically investigating whether and how it influences the disease process.

## Results

### Impaired CMA in a murine model of IMQ-induced psoriasiform dermatitis

**Figure 1.**
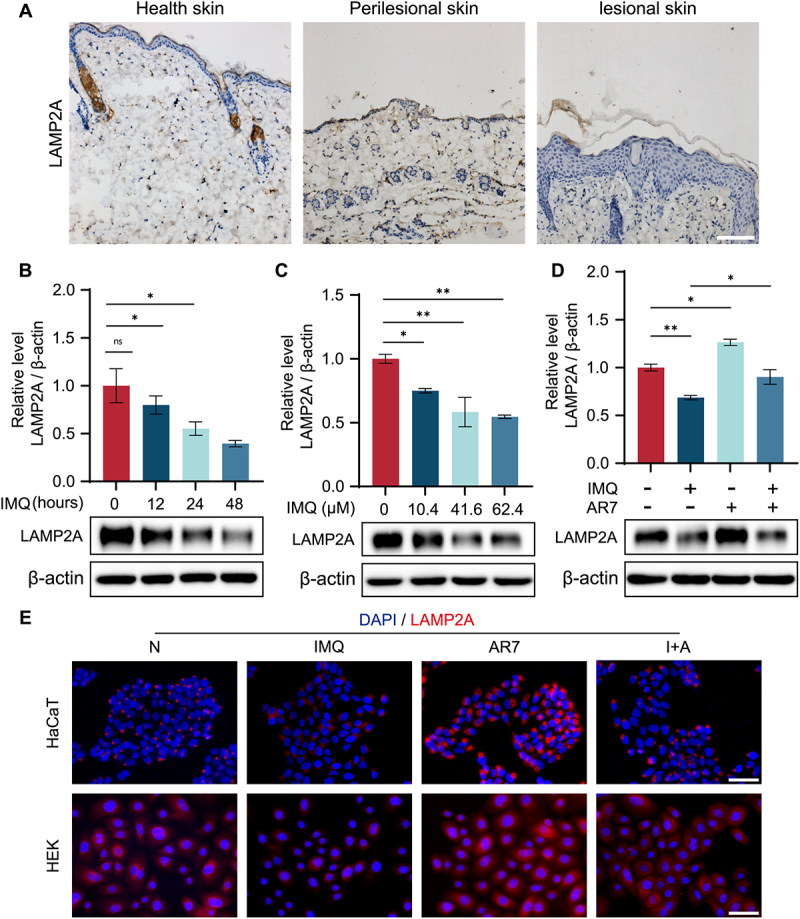
Changes in LAMP2A expression after treatment with IMQ, AR7, or both *in vivo* and *in vitro*. (A) LAMP2A immunohistochemistry in mouse epidermis (normal, IMQ-induced perilesional, and lesional (scale bar = 100 μm). (B) Western blot analysis of LAMP2A expression in HaCaT cells treated with or without IMQ (41.6 μM) for 12–48 h. The bars present the average values from three independent experiments plus SD. *p < 0.05. (C) Western blot analysis of LAMP2A expression in HaCaT cells treated with varying concentrations of IMQ for 24 h. The bars present the average values from three independent experiments plus SD. *p < 0.05, **p < 0.01. (D) LAMP2A expression in HaCaT cells treated with IMQ (41.6 μM) and (or) AR7 (20.0 μM) for 24 h. The bars present the average values from three independent experiments plus SD. *p < 0.05, **p < 0.01. (E) Immunofluorescence analysis of LAMP2A expression in HaCaT and HEK cells treated with IMQ (41.6 μM) and (or) AR7 (20.0 μM) for 24 h (scale bar = 100 μm).

To determine the CMA activity in psoriatic lesions, we analyzed the expression of LAMP2A, a crucial regulator of CMA, in a mouse model of IMQ-induced psoriasis-like skin inflammation. Our findings showed that LAMP2A expression was significantly lower in lesional skin compared to healthy control epidermis (Figure 1A). Given the critical role of keratinocytes in psoriasis, we further used IMQ-treated keratinocytes to assess the involvement of CMA in these cells. As shown in Figure 1B,C, LAMP2A levels decreased in HaCaT cells treated with IMQ in a time- and concentration-dependent manner, demonstrating that IMQ suppresses CMA activity in keratinocytes. In contrast, treatment with the classical CMA activator AR7 dramatically increased LAMP2A expression in both IMQ-untreated and IMQ-treated HaCaT cells (Figure 1D). Furthermore, immunofluorescence analysis showed that AR7 reversed the IMQ-induced decrease in CMA activity in both HaCaT and HEK cells (Figure 1E).

### CMA regulation of keratinocyte proliferation and differentiation in response to IMQ

**Figure 2.**
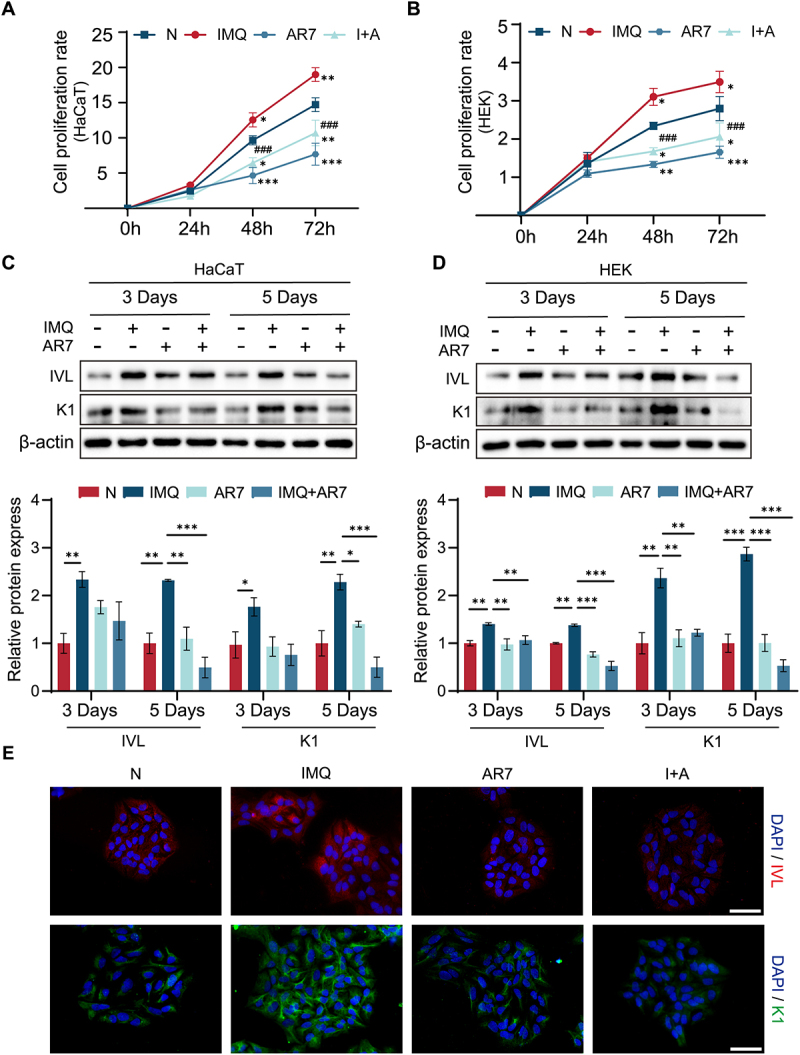
The effect of CMA on IMQ-induced keratinocyte proliferation and differentiation. (A, B) HaCaT and HEK cells proliferation in response to IMQ (4.2 μM) and (or) AR7 (20.0 μM) treatment (24–72 h), assessed by CCK-8 assay. The bars present the average values from three independent experiments plus SD. *p < 0.05, **p < 0.01, and ***p < 0.001, *vs* the non-treated (N) group; ^###^*p* < 0.001, *vs* the IMQ-treated group. (C, D) Involucrin (IVL) and cytokeratin 1 (K1) expression was detected by immunoblotting (top panel) and quantified (bottom panel) in HaCaT and HEK cells after 5 days in KGM, followed by treatment with IMQ (10.4 μM) and (or) AR7 (10.0 μM) for 3 or 5 days. The bars present the average values from three independent experiments plus SD. *p < 0.05, **p < 0.01, and ***p < 0.001. (E) Immunofluorescence staining of IVL and K1 in HaCaT and HEK cells treated with IMQ (10.4 μM) and (or) AR7 (10.0 μM) for 5 days after 5 days in KGM (scale bars = 100 μm).

IMQ is widely used to induce psoriasiform lesions in mouse skin, as it alters keratinocyte proliferation and differentiation. To investigate the role of CMA in these processes, we examined its involvement in IMQ-induced keratinocyte proliferation and differentiation. HaCaT and HEK cells were treated with IMQ, with or without AR7, for 24, 48, and 72 h. While IMQ treatment for 48 and 72 h significantly promoted cell growth compared to control conditions, subsequent AR7 treatment significantly inhibited this IMQ-induced cell proliferation (Figure 2A,B). Additionally, IMQ-induced cell proliferation was dramatically inhibited by LAMP2A overexpression (Fig. S1A,B).

To maintain their growth and undifferentiated status, keratinocytes were routinely cultured in the keratinocyte growth medium (KGM) lacking Ca^2+^. Following this, IMQ and AR7 were used to stimulate the HaCaT and HEK cells and induce differentiation. The differentiation of keratinocytes from the basal to cornified layers of the epidermis is characterized by a temporal progression of molecular marker expression. Cytokeratins 1 and 10 appear earlier in the spinous and granular layers, while involucrin marks the final stages of differentiation in the granular layer and cornified layer. Western blotting results showed that IMQ stimulation significantly increased involucrin and cytokeratin 1 expression in HaCaT and HEK cells, reaching peak levels on day 5. However, treatment with AR7 alone or in combination with IMQ significantly reduced the expression of involucrin and cytokeratin 1 on day 5 compared to the IMQ-only group (Figure 2C,D). Moreover, LAMP2A overexpression significantly inhibited IMQ-promoted cell differentiation (Fig. S1C). Immunofluorescence analysis corroborated these findings (Figure 2E), showing increased involucrin and cytokeratin 1 positivity in HaCaT cells following IMQ treatment compared to the control. Again, AR7 treatment, either alone or in combination with IMQ, significantly reduced involucrin and cytokeratin 1 expression compared to the IMQ-only group. Therefore, CMA appears to negatively regulate IMQ-induced keratinocyte proliferation and differentiation.

### Enhanced CMA mitigates IMQ-induced psoriasiform lesions

**Figure 3.**
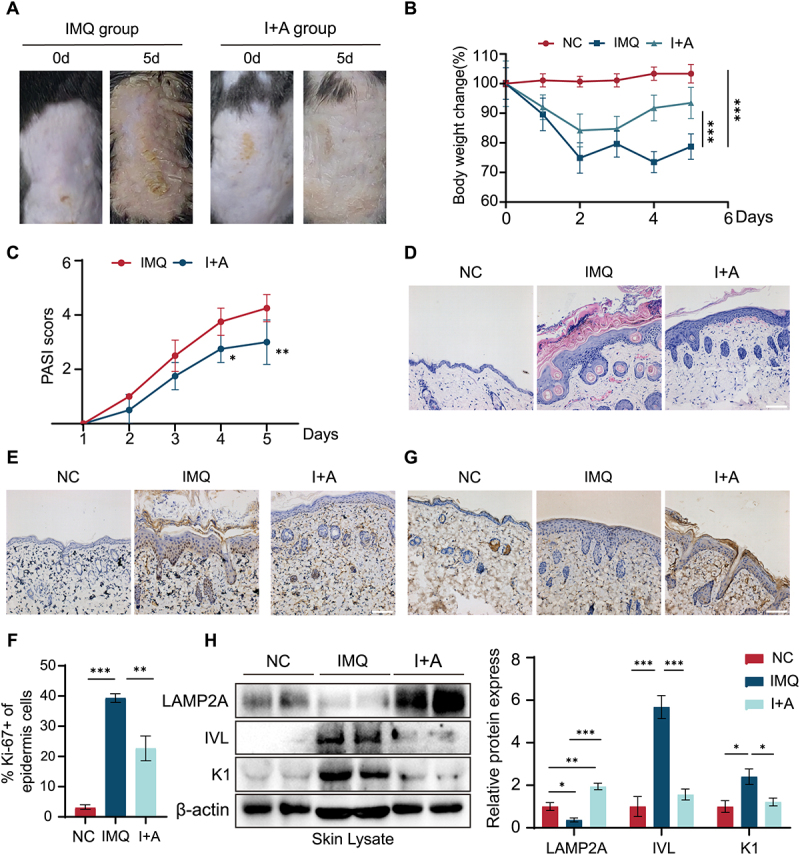
Enhanced CMA mitigates IMQ-induced psoriasiform lesions. (A) Day 5 dorsal skin: representative images from each treatment group. (B) 5-day body weight changes in mice (*n* = 4/group). The bars present the average values from four mice plus SD. ***p < 0.001. (C) PASI scores for each treatment group (*n* = 4/group). The bars present the average values from four mice plus SD. *p < 0.05, **p < 0.01. (D) Representative H&E-stained histological sections of mouse dorsal skin (day 5) (scale bar = 100 μm). (E) Ki-67 staining in mice dorsal skin on day 5 (scale bar = 100 μm). (F) Quantitative analysis of Ki-67-positive cells in mice dorsal skin on day 5 (*n* = 4/group). The bars present the average values from four mice plus SD. **p < 0.01, ***p < 0.001. (G) LAMP2A immunostaining of mouse dorsal skin on day 5 (scale bar = 100 μm). (H) Western blot analysis of skin lysates for LAMP2A, involucrin (IVL), and cytokeratin 1 (K1) expression. Quantitative analysis of LAMP2, IVL, and K1 protein expression relative to β-actin (*n* = 4 per group). The bars present the average values from three independent experiments plus SD. *p < 0.05, **p < 0.01, ***p < 0.001. NC: normal skin; IMQ: IMQ-induced lesions; I+A: IMQ lesions+AR7 (i.P.).

To investigate the role of CMA dysregulation in IMQ-induced psoriasis-like lesions *in vivo*, we applied IMQ cream daily for 5 days to the shaved dorsal skin of mice, inducing psoriasiform lesions. To explore the potential *in vivo* antipsoriatic effects of CMA activation, we administered intraperitoneal injections of AR7 to these mice once every other day (Fig. S2). The dorsal area of mice treated with IMQ gradually exhibited erythema, scaling, and skin thickening, which progressively increased in severity from days 2 to 5 of the experiment. However, these symptoms were dramatically mitigated in mice injected with AR7 (Figure 3A). Considering that psoriasis involves immune system dysfunction, leading to systemic damage and potential weight loss, we monitored the weight of the mice. Within days 2–3 after the initiation of the IMQ-induced psoriasis model, the weight of mice in all groups except the control group significantly decreased. This weight loss was subsequently reversed, gradually increasing in subsequent days. Interestingly, a more significant body weight gain was observed in the (IMQ+AR7)-treated group compared to the IMQ-treated group on days 4–5 (Figure 3B). The Psoriasis Area and Severity Index (PASI) scores in the IMQ-treated group dramatically increased from day 1 to day 5. By contrast, the (IMQ+AR7)-treated group showed a significant reduction in PASI scores (Figure 3C).

Histological examination of H&E-stained sections from skin treated with IMQ for 5 days revealed characteristic psoriasis-like changes, including epidermal parakeratosis, severe capillary congestion, and significant epidermal thickening (Figure 3D). Analysis of epidermal thickness revealed a significant increase in the IMQ-treated group compared to the control group. The (IMQ+AR7)-treated group exhibited a statistically significant decrease in epidermal thickness compared to the IMQ-treated group. Then, nuclear protein Ki-67 immunostaining was performed to examine the keratinocyte proliferation in the epidermis of IMQ-treated mice. As shown in Figure 3E,F, there was a significant increase in the number of Ki-67-positive cells in the epidermis of the IMQ-treated group compared with normal mice. However, AR7 treatment significantly reduced this increase in proliferating keratinocytes.

To detect CMA activity in IMQ-induced psoriasiform lesions, we examined LAMP2A expression in the epidermis of skin sections from mice. The IHC analysis showed a higher expression of LAMP2A in the epidermis of (IMQ+AR7)-treated mice compared to mice treated with IMQ alone (Figure 3G). Western blot analysis of skin lysates from IMQ-treated mice showed a significant reduction in LAMP2A expression and a significant increase in both involucrin and cytokeratin 1 expression compared to controls (Figure 3H). Conversely, the (IMQ+AR7)-treated group exhibited a substantial increase in LAMP2A protein expression and a significant decrease in involucrin and cytokeratin 1 protein expression. The findings of IHC analysis for involucrin and cytokeratin 1 were in agreement with the results obtained by Western blot analysis (Fig. S3). Taken together, *in vivo* studies indicate that CMA activation has the potential to ameliorate IMQ-induced psoriasis-like skin lesions by modulating keratinocyte proliferation and differentiation.

### CMA regulation of cytokine production in IMQ-induced psoriasis

**Figure 4.**
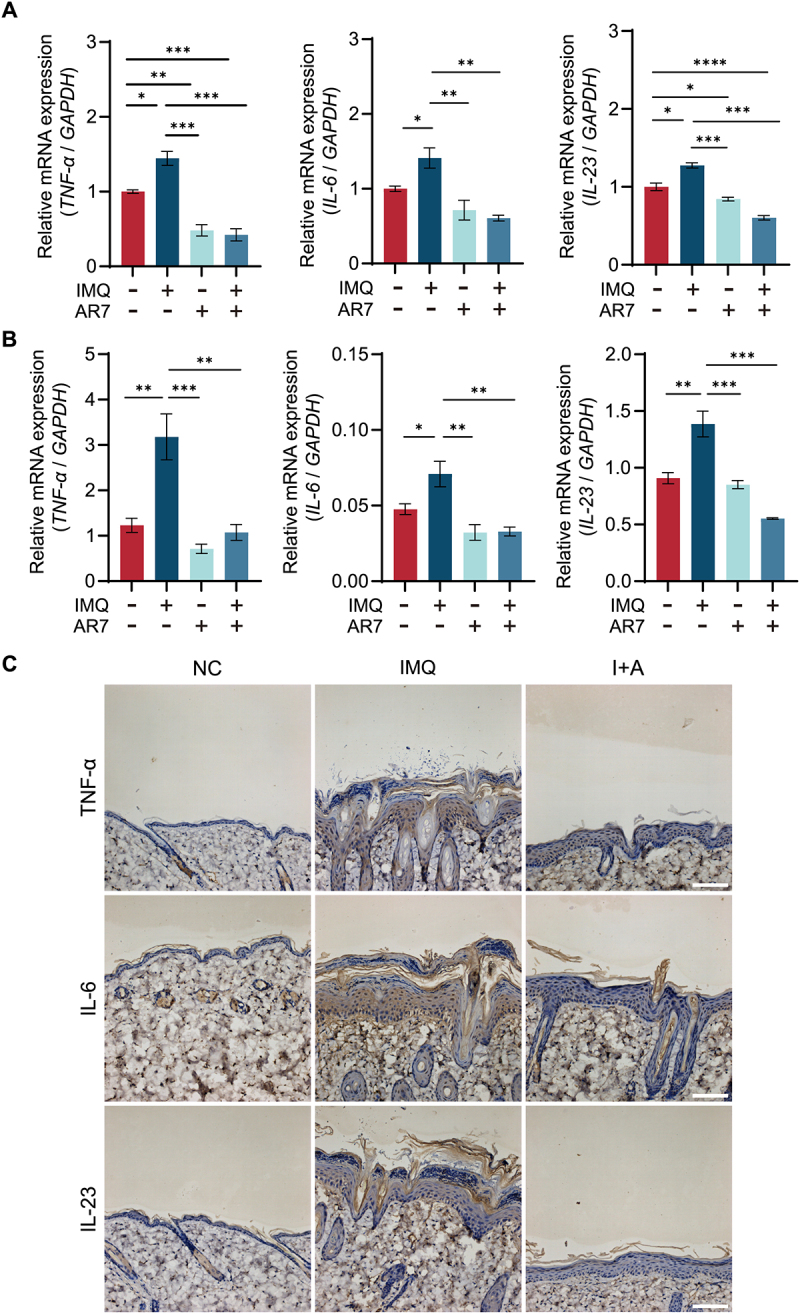
CMA regulates IMQ-induced productions of cytokines. (A) Quantitative RT-PCR analysis of *TNF-α*, *IL-6*, and *IL-23* mRNA expression in HaCaT cells treated with IMQ (41.6 μM) and (or) AR7 (20.0 μM) for 24 h. The bars present the average values from three independent experiments plus SD. *p < 0.05, **p < 0.01, ***p < 0.001, and ****p < 0.0001. (B) Quantitative RT-PCR analysis of *TNF-α*, *IL-6*, and *IL-23* mRNA expression in HEK cells treated with IMQ (41.6 μM) and (or) AR7 (20.0 μM) for 24 h. The bars present the average values from three independent experiments plus SD. *p < 0.05, **p < 0.01, and ***p < 0.001. (C) Immunohistochemistry analysis of TNF-α, IL-6, and IL-23 protein expression in mouse dorsal skin (scale bar = 100 μm).

Secreted cytokines from keratinocytes and immune cells are also thought to play a major role in the inflammatory loop of psoriasis. To investigate the effect of CMA on the expression of proinflammatory cytokines involved in keratinocytes, we examined the mRNA levels of *TNF-α*, *IL-6*, and *IL-23* in HaCaT and HEK cells treated with IMQ and AR7. Our results demonstrated that IMQ significantly upregulated the mRNA levels of these cytokines, while treatment with AR7 significantly suppressed their expression (Figure 4A,B). Additionally, qRT-PCR analysis of skin lesions in mice revealed significantly elevated mRNA levels of *Tnf-α*, *Il-6*, and *Il-23* in the IMQ-treated group compared to normal controls. In contrast, the (IMQ+AR7)-treated group exhibited significant downregulation of these cytokine mRNA levels (Fig. S4). Furthermore, IHC analysis results corroborated those of the qRT-PCR analysis (Figure 4C). These data suggest that CMA activation inhibits IMQ-induced production of cytokines.

### CMA-mediated TLR7 degradation and NF-κB activation in IMQ-induced psoriasis

**Figure 5.**
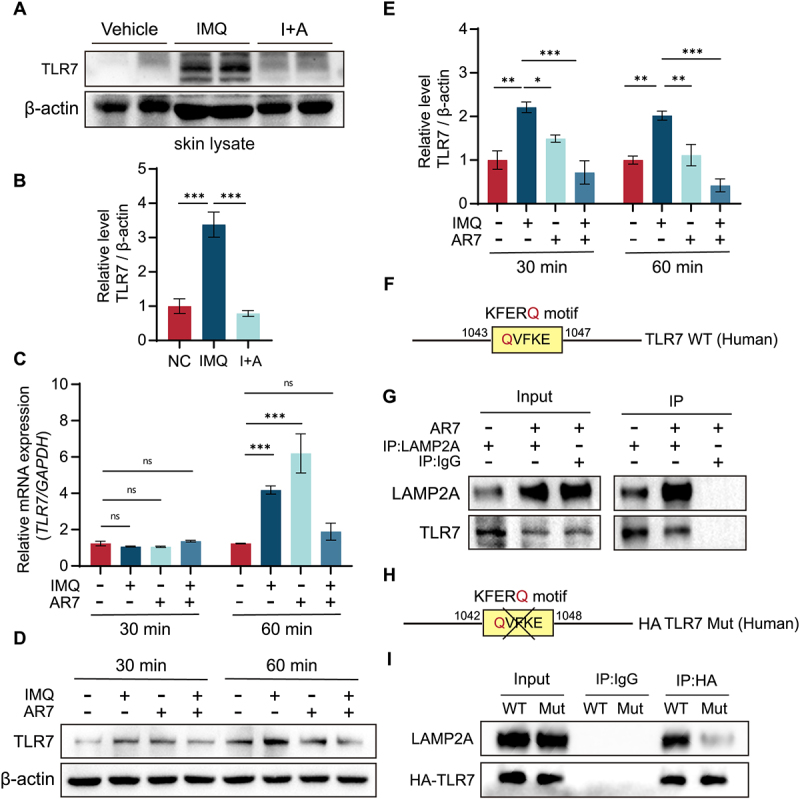
CMA-dependent TLR7 degradation is involved in the IMQ-induced cell response. (A) Western blot analysis of skin lysates for TLR7 protein expression. (B) Quantitative analysis of TLR7 protein expression relative to β-actin. The bars present the average values from three independent experiments plus SD. ***p < 0.001. (C) Time-course analysis of *TLR7* mRNA expression in HaCaT cells treated with IMQ (41.6 μM) and (or) AR7 (20.0 μM). The bars present the average values from three independent experiments plus SD. ns: no significant; ***p < 0.001. (D) Time-course analysis of TLR7 protein expression in HaCaT cells treated with IMQ (41.6 μM) and (or) AR7 (20.0 μM). (E) Quantitative analysis of TLR7 protein expression. The bars present the average values from three independent experiments plus SD. *p < 0.05, **p < 0.01, and ***p < 0.001. (F) Analysis of a KFERQ-like motif (QVFKE) in the human TLR7 amino acid sequence. (G) Co-immunoprecipitation of LAMP2A and TLR7 in HaCaT cells: effect of AR7 treatment (20.0 μM). Rabbit IgG served as a negative control. (H) Schematic of the QVFKE deletion (KFERQ-like motif) in the human TLR7 amino acid sequence. (I) Co-immunoprecipitation of LAMP2A with mutated TLR7 in HaCaT cells. HaCaT cells were transfected with HA-tagged human *TLR7* cDNA, lacking the QVFKE motif. Immunoprecipitation was performed using an anti-HA antibody. Rabbit IgG served as a negative control.

IMQ, which acts as a TLR7 agonist, activates the TLR7 signaling pathway, a crucial factor in IMQ-induced psoriasis-like skin inflammation and, equally important, in regulating keratinocyte proliferation and differentiation (Fig. S5). While various TLRs are implicated in psoriasis, only TLR7 expression was dramatically increased in keratinocytes following IMQ treatment (Fig. S6). Western blot analysis of skin lysates from mice with IMQ-induced psoriatic lesions further revealed a significant increase in TLR7 protein expression compared to lysates from normal mouse epidermis. However, treatment with AR7 effectively inhibited this increase in TLR7 expression in skin lysates from psoriasiform lesions (Figure 5A,B). To further investigate TLR7 expression at the cellular level, we treated HaCaT cells with IMQ and AR7. While we observed no significant changes in *TLR7* mRNA levels in keratinocytes treated with IMQ or AR7 for 30 min, a significant increase in *TLR7* mRNA levels was also observed when keratinocytes were treated with IMQ or AR7 alone for 60 min compared to the control group (Figure 5C). However, this increase in *TLR7* mRNA levels was significantly restored to the level of the normal group when IMQ and AR7 were combined. Western blot analysis showed that a significantly increased level of TLR7 was observed in HaCaT cells treated with IMQ. Importantly, this increase of TLR7 was dramatically inhibited by AR7 treatment (Figure 5D,E). These findings provide evidence that CMA-mediated degradation may play a role in regulating TLR7 protein expression in response to IMQ and AR7.

We further analyzed the primary structure of TLR7 to determine if it is a direct target for CMA-mediated degradation. Sequence analysis revealed that TLR7 contains a conserved QVFKE motif at residues 1043–1047 (Figure 5F), which is a canonical KFERQ-like motif recognized by HSC70 and specifically binds to LAMP2A, a key component of CMA-mediated degradation. This suggests that TLR7 may be a potential substrate for CMA. Notably, co-immunoprecipitation analysis confirmed the specific interaction between TLR7 and LAMP2A (Figure 5G). Deletion of the QVFKE motif in TLR7 abolished this specific interaction with LAMP2A (Figure 5H,I). These findings demonstrate that increased TLR7 levels promote IMQ-induced psoriasis-like skin inflammation and lesions, likely due to a decrease in TLR7 degradation caused by CMA inhibition.

**Figure 6.**
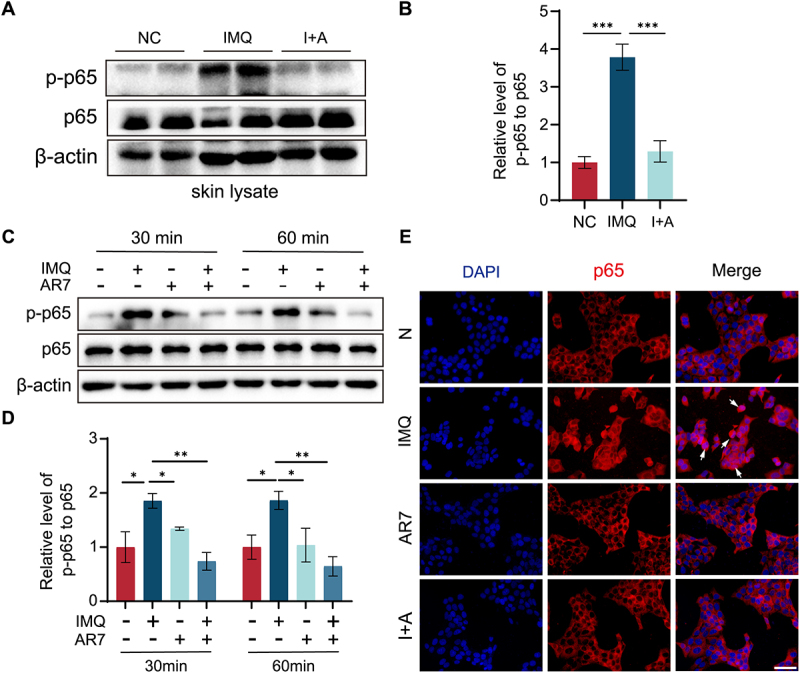
CMA regulates IMQ-reduced NF-κB transcriptional activity. (A) Western blot analysis of p-p65 in mouse skin lysates. (B) Quantification of the p-p65 relative to p65. The bars present the average values from three independent experiments plus SD. ***p < 0.001. (C) Western blot analysis of p-p65 protein levels in HaCaT cells treated with IMQ (41.6 μM) and (or) AR7 (20.0 μM) for 30 and 60 min. (D) Quantitative analysis of the p-p65:p65 ratio from panel C. The bars present the average values from three independent experiments plus SD. *p < 0.05, **p < 0.01. (E) Immunofluorescence analysis of p65 localization in HaCaT cells after 60-min treatment with IMQ (41.6 μM) and (or) AR7 (20.0 μM). Scale bar = 100 μm. Arrows indicate p65 nuclear translocation in IMQ-treated HaCaT cells.

Given that IMQ-induced psoriasis-like skin inflammation and lesions are associated with activation of the TLR7-NF-κB (p65) signal, we then investigated whether CMA modulates IMQ-induced NF-κB activation in psoriasiform lesions. As shown in Figure 6A,B, IMQ treatment significantly increased phosphorylated-p65 (p-p65) levels in total skin lysates, while AR7 treatment significantly reduced these levels in IMQ-treated mice compared to IMQ alone. In HaCaT cells, both 30 and 60 min of IMQ treatment significantly increased p-p65 levels, an effect significantly inhibited by AR7 (Figure 6C,D). Immunofluorescence analysis showed that IMQ treatment induced p65 nuclear translocation in HaCaT cells, while AR7 treatment prevented this translocation, consistent with its effect on NF-κB activation (Figure 6E). These findings indicate that CMA activation leads to TLR7 degradation and subsequent inhibition of TLR7-NF-κB (p65) activation in keratinocytes.

## Discussion

Autophagy defects are consistently associated with diverse skin disorders, including infectious diseases, skin cancers, and inflammatory or autoimmune conditions like psoriasis, systemic lupus erythematosus, and atopic dermatitis^[32,33]^. While the precise role of autophagy in psoriasis pathogenesis remains debated due to its varied effects across cell types (lymphocytes, keratinocytes, monocytes, mesenchymal stem cells)^[13]^, evidence of dysfunctional autophagy in keratinocytes mediating proliferation, differentiation, and cytokine production strongly implicates it in the disease process. CMA, a specific type of autophagy, plays a critical role in cellular homeostasis, particularly during stress responses, by selectively degrading specific proteins. While CMA’s precise role in regulating various cellular processes is well-established, its potential involvement in the development and progression of certain inflammatory diseases is an area of active research. For example, in atherosclerosis, impaired CMA function leads to increased NOD-like receptor family pyrin domain containing three inflammasome activation and IL-1β secretion, promoting vascular inflammation and disease progression^[22,34–36]^. Conversely, CMA activation protects chondrocytes from damage in joint aging and osteoarthritis^[37]^. In nonalcoholic steatohepatitis, deficient CMA in macrophages aggravates inflammation^[38]^. Furthermore, CMA represses microglial activation through the p300-associated NF-κB (p65) signaling pathway, demonstrating a direct link between CMA and neuroinflammation^[39]^. While the role of CMA in these inflammatory diseases is increasingly clear, its function in psoriasis remains less understood.

This study provides the first evidence of defective CMA in IMQ-induced psoriasiform lesions in mice, suggesting a potential role for CMA dysfunction in the development and progression of psoriasis. Keratinocytes treated with IMQ also exhibit a decrease in LAMP2A expression, indicating that IMQ inhibits CMA. Importantly, AR7, a specific activator of CMA, dramatically attenuates IMQ-induced psoriasiform lesions, resulting in a reduction of erythema, scaling, and skin thickening, as well as an increase in mouse weight. At the cellular level, AR7 also activates CMA, effectively reversing the IMQ-induced proliferation, differentiation, and cytokine production of keratinocytes. These findings suggest a mechanistic link between defective CMA and the development of psoriasis, highlighting a potential therapeutic target for treating this chronic inflammatory skin disease.

While IMQ-induced macroautophagy in macrophages is dependent on the activation of double-stranded RNA-dependent protein kinase, triggered by reactive oxygen species-mediated endoplasmic reticulum stress^[40,41]^, the presence of a KFERQ-like motif in TLR7, the IMQ-targeted activation receptor, identifies it as a target substrate for CMA-mediated degradation. Defective CMA in psoriatic keratinocytes leads to increased levels of TLR7, resulting in the activation of TLR7 signaling. This, in turn, recruits myeloid differentiation factor 88 and activates the NF-κB pathway, which has been identified as a mediator of inflammatory responses in psoriatic lesions^[42–46]^. In our study, activation of CMA contributes to a reduction in NF-κB (p65) phosphorylation, both in IMQ-induced psoriasiform lesions and in keratinocytes treated with IMQ. Furthermore, AR7 treatment inhibits IMQ-induced nuclear translocation of NF-κB (p65) in HaCaT cells. These findings demonstrate an alternative mechanism for TLR7 degradation via CMA and reveal a novel role for CMA in regulating NF-κB signaling, underscoring its importance in the pathogenesis of psoriasis.

## Conclusion

Despite the focus on a single animal model and cell culture models, this study revealed several key findings: First, defective CMA was observed in IMQ-induced psoriasiform lesions and IMQ-treated keratinocytes; Second, activation of CMA significantly mitigated IMQ-induced psoriasiform lesions; Third, activation of CMA in keratinocytes benefited for the regulation of keratinocyte proliferation, differentiation, and cytokine secretion; Forth, CMA-mediated degradation of TLR7 contributed to the inhibition of the TLR7-NF-κB signaling pathway implicated in psoriasis. These findings suggest that pharmacological activation of CMA may represent a promising and novel therapeutic strategy for psoriasis.

## Materials and methods

### Reagents

A complete list of the reagents used in this study is provided in Table 1. Antibody information is available in Table 2. The nucleotide sequences (Table S1) were obtained from the Sangon Biotech Company (Shanghai, China).

**Table 1.** Reagent list.

Reagents	Company (City, Country)	Cat no.
Dulbec’s Modified Eagle Medium (DMEM)	Gibco (Grand Island, NY, USA)	11995500Bt
Fetal Bovine Serum (FBS)	ExCell (Suzhou, China)	FSP500
Keratinocyte growth medium (KGM)	Gibco (Grand Island, NY, USA)	M154CF500
7-chloro-3-(4-methylphenyl)-2 H–1,4-benzoxazine (AR7)	Med Chem Express (Shanghai, China)	HY-101106
Enpatoran (M5049)	Med Chem Express (Shanghai, China)	HY-134581
Imiquimod (IMQ) powder (Dissolved in water)	Invitrogen (Carlsbad, CA, USA)	R837
IMQ cream (5%)	Sichuan Ming Xin Pharmaceutical Co, LTD (Chengdu, China)	H20030128
RIPA lysis buffer	Biosharp (Hefei, China)	BL504A
ECL reagents	Advansta (California, USA)	K-12045-D50
IP Cell Lysis Solution	Beyotime Biotechnology (Shanghai, China)	P0013
Protein G Agarose Beads	Cell Signaling Technology (Danvers, MA, USA)	37478
DAPI	Beyotime Biotechnology (Shanghai, China)	P0131
Cell Counting Kit-8 (CCK-8)	Abbkine (Wuhan, China)	KTA1020
Trizol	Invitrogen (Carlsbad, CA, USA)	15596026
All-in-one RT Easy Mix for qPCR	Tolobio (Anhui, China)	22107
2×Q3 SYBR qPCR Master Mix (Universal)	Tolobio (Anhui, China)	22204
3,3ʹ-diaminobenzidine (DAB) substrate kit	ZSGB-BIO (Beijing, China)	ZLI-9017

**Table 2.** Antibody list.

Antibody	Company (City, Country)	Cat. no.	Use	Dilution
Anti-LAMP2A	Huabio (Hangzhou, China)	ET1601–24	^1^WB; ^2^CO-IP; ^3^IHC	WB(1:4000); CO-IP(1:100); IHC(1:400)
Anti-LAMP2A	Beyotime Biotechnology (Shanghai, China)	AF1036	^4^IF	1:200
Rabbit (DA1E) mAb IgG XP isotype control	Cell Signaling Technology (Danvers, MA, USA)	3900	^5^IP	1:100
Anti-cytokeratin-1	Proteintech (Wuhan, China)	16848–1-AP	WB	1:1000
Anti-TLR7	Proteintech (Wuhan, China)	17232–1-AP	WB	1:1000
Anti-involucrin	Proteintech (Wuhan, China)	28462–1-AP	WB	1:1000
Anti-NF-κB p65	Santa Cruz Biotechnology (CA, USA)	SC-8008	WB	1:500
Anti-phospho-NF-κB p65	Santa Cruz Biotechnology (CA, USA)	SC-136548	WB	1:500
Anti-involucrin	Santa Cruz Biotechnology (CA, USA)	SC-21748	IF	1:200
Anti-cytokeratin-1	Proteintech (Wuhan, China)	16848–1-AP	IF	1:200
Anti-NF-κB p65	Santa Cruz Biotechnology (CA, USA)	SC-8008	IF	1:200
Anti-cytokeratin-1	Proteintech (Wuhan, China)	16848–1-AP	IF	1:200
Anti-MKi-67	Huabio (Hangzhou, China)	HA721115	IHC	1:200
Anti-IL-6	Huabio (Hangzhou, China)	R1412–2	IHC	1:50
Anti-involucrin	Proteintech (Wuhan, China)	28462–1-AP	IHC	1:100
Anti-cytokeratin-1	Proteintech (Wuhan, China)	16848–1-AP	IHC	1:200
Anti-TNF-α	ZEN-BIOSCIENCE (Chengdu, China)	346654	IHC	1:50
Anti-IL-23	Abcam (Cambridge, MA, USA)	ab115759	IHC	1:50
Anti-mouse IgG, HRP-linked antibody	Cell Signaling Technology (Danvers, MA, USA)	7076	WB	1:2000
Anti-rabbit IgG, HRP-linked antibody	Cell Signaling Technology (Danvers, MA, USA)	7074	WB	1:2000
Anti-Rabbit IgG Fab2 Alexa Fluor (R) 594	Cell Signaling Technology (Danvers, MA, USA)	8889 S	IF	1:500
Anti-Mouse IgG Fab2 Alexa Fluor (R) 488	Cell Signaling Technology (Danvers, MA, USA)	4408 S	IF	1:500
Anti-HA antibody	Proteintech (Wuhan, China)	51064–2-AP	IP	1:100
Anti-TLR4 antibody	Affinity Biosciences	#AF-7017	WB	1:500
Anti-TLR2 antibody	Affinity Biosciences	#DF-7521	WB	1:1000
Anti-TLR3 antibody	Affinity Biosciences	#DF-6415	WB	1:500
Anti-TLR7 antibody	Affinity Biosciences	#DF-6173	WB	1:500
Anti-β-actin	Proteintech (Wuhan, China)	66009–1-Ig	WB	1:1000

^a^WB:Western blot; ^2^CO-IP:Co-immunoprecipitation; ^3^IHC: Immunohistochemistry; ^4^IF: Immunofluorescence; ^5^IP: Immunoprecipitation.

### Experimental animals

Eight-week-old male C57BL/6J mice were obtained from Gem Pharmatech Co. (Nanjing, China) and housed under specific pathogen-free conditions with a 12-h light/dark cycle at 25°C, with normal food and water. To induce psoriasis, four randomly selected mice (two males and two females) received daily topical applications of 60 mg of 5% IMQ cream to a 4 cm^2^ shaved area on their dorsal skin for 5 consecutive days.

To assess the effects of AR7 (a benzoxazine derivative and CMA activator) on IMQ-induced psoriasiform lesions, a 4 cm^2^ area on the backs of all mice was shaved one day prior to the start of treatment (day −1). The mice were then randomly assigned to one of the three groups (*n* = 4, equally divided by sex) and received the following treatments (Fig. S2): (1) the normal control (NC) group received standard chow and water; (2) the IMQ-treated group received intraperitoneal (i.p.) injections of 0.5% dimethyl sulfoxide in saline on days 0, 2, and 4 (IMQ group); and (3) the (IMQ+AR7)-treated group received the IMQ treatment described above, plus i.p. injections of 5 mg/kg AR7 on days 0, 2, and 4 (I+A group).

Mice were euthanized on day 5, and samples were collected for subsequent analysis. From days 1 to 5, psoriasis-like lesion severity was monitored using the PASI scores, which assess erythema, scaling, and thickness on a 0–4 scale (0 = none, 1 = slight, 2 = moderate, 3 = marked, 4 = very marked)^[47]^.

### Cell culture

The immortalized human keratinocyte cell line (HaCaT) was obtained from the National Biomedical Experimental Cell Resource Bank (Beijing, China) and cultured in DMEM supplemented with 10% FBS and 1% penicillin−streptomycin (37°C, 5% CO_2_). Primary human keratinocytes (HEK) at passage 1 were purchased from Wanwu Biotechnology (Hefei, China). Cells were cultured in the Keratinocyte Growth Medium (KGM) supplemented with growth additives and 1% penicillin-streptomycin at 37°C in a 5% CO_2_ atmosphere for two to three passages. To assess proliferation, cells were treated with IMQ (4.2 μM) and (or) AR7 (a benzoxazine derivative) (20.0 μM) for 24–72 h. For differentiation studies, cells were cultured in KGM for 5 days before treatment with IMQ (10.4 μM) and (or) AR7 (10.0 μM) for 3 or 5 days. In other experiments, cells were treated with IMQ (41.6 μM) and AR7 (20.0 μM) over a defined time course.

### Immunoblotting analysis

Cell and skin lysate proteins were resolved by SDS-PAGE, transferred to PVDF membranes, and subjected to immunoblotting^[29]^. Immunoblot band intensities were quantified using ImageJ, and statistical analysis and graphing were performed using GraphPad Prism 8.4.0.

### Co-immunoprecipitation

Cells were lysed in Immunoprecipitation Cell Lysis Solution, centrifuged, and the supernatant was incubated overnight at 4°C with a primary antibody conjugated to protein G agarose. After five washes with lysis buffer, immunoprecipitates and input samples were resolved by SDS-PAGE and analyzed by Western blotting.

### Immunofluorescence staining

Immunofluorescence staining was performed as previously described^[48]^. Briefly, cells were fixed with 4% paraformaldehyde, permeabilized with 0.2% Triton X-100, washed with PBS, and blocked with 10% horse serum. Primary and secondary antibodies were diluted in blocking buffer, and the images were captured using a DM6 B microscope (Leica, Germany).

### Hematoxylin and eosin (H&E) staining

Mouse dorsal skin was fixed in 4% paraformaldehyde for at least 24 h, paraffin-embedded, and sectioned at 4 μm. Sections were then stained with H&E for histopathological examination. Epithelial thickness was measured using a TissueFAX Plus ST (Tissue Gnostics, Austria).

### Immunohistochemistry

Immunohistochemistry was performed as previously described^[29,48]^. Briefly, sections were dewaxed, rehydrated, and treated with 3% hydrogen peroxide to quench endogenous peroxidases. Antigen retrieval was performed by microwaving in 1% citrate buffer for 15 min, followed by blocking with 1.5% blocking serum. Sections were incubated with primary antibodies, and then immunostaining was completed using biotinylated anti-IgG, DAB detection, and hematoxylin counterstaining. Imaging was performed using a TissueFAX Plus ST system (Tissue Gnostics, Austria).

### Cell viability assay

Cell viability was determined using a Cell Counting Kit-8 (CCK-8) assay. Following drug treatment (specified concentrations and times), cells plated in 96-well plates were measured at 450 nm using an EnSpire multimode reader (PerkinElmer, USA).

### Quantitative real-time RT-PCR

Following RNA extraction from cells or tissues, cDNA synthesis was performed using All-in-one RT Easy Mix. Quantitative real-time PCR (qPCR) was subsequently performed using SYBR Green and primers on a LightCycler 480 II system (Roche Life Science, Switzerland).

### Statistical analysis

Data, representing the mean ± SD from at least three independent experiments, were analyzed using one-way or two-way ANOVA followed by Tukey’s post hoc test for multiple comparisons. A *p* value < 0.05 was considered statistically significant.

## Abbreviations

AR7: atypical retinoid 7
CMA: chaperone-mediated autophagy
HSC70: heat shock protein family A (Hsp70) member 8
IHC: immunohistochemistry
IL: interleukin
IMQ: imiquimod
KGM: keratinocyte growth medium
Ki-67: nuclear proliferation-associated antigen MKI-67 protein
LAMP2A: lysosome-associated membrane protein type 2A
NF-κB: nuclear factor kappa B
PASI: psoriasis area and severity index
TLR7: toll-like receptor 7
TNF-α: tumor necrosis factor-α.

## Supplementary Material

SUPPLEMENTARY DATA for submission_Clean.docx

## Data Availability

The datasets generated during this study are fully available within this article and the supplementary materials.

## Study approval

All animal experiments were conducted following protocols approved by the Institutional Animal Care and Use Committee of Anhui Medical University (Approval No. LLSC20200007).

## Notes

The authors disclose no use of AI in the writing of this manuscript.
