# Supplementary material for: Chaperone-mediated autophagy dysfunction in imiquimod-induced psoriasiform dermatitis
Source: Autophagy Rep. 2025 Aug 25;4(1):2544061. doi: 10.1080/27694127.2025.2544061 (PMC12380211; doi:10.1080/27694127.2025.2544061)
Supplement: SUPPLEMENTARY DATA for submission_Clean.docx [file KAUO_A_2544061_SM4426.docx]

***Supplementary Information***

Chaperone-Mediated Autophagy Dysfunction in Imiquimod-Induced Psoriasiform Dermatitis

Wei Zhao ^a, #^, Kainan Liao ^a, #^, Wei Song ^a^, Jing Wang ^a^, Chunlin Cai ^b^, Fusheng Zhou ^c^ Dandan Zang ^d^, Deping Xu ^e, *^, Haisheng Zhou^a, c, *^

^a^ Department of Biochemistry and Molecular Biology, School of Basic Medical Sciences of Anhui Medical University, Hefei, China.

^b^ Department of Pathophysiology, School of Basic Medical Sciences, Anhui Medical University, Hefei, China.

^c^ Key Laboratory of Dermatology, Anhui Medical University & Ministry of Education, Hefei, China.

^d^ Center for Scientific Research, Anhui Medical University. Hefei, China.

^e^ Clinical Laboratory, The second People’s Hospital of Hefei, Hefei Hospital Affiliated to Anhui Medical University, Hefei, China.

^#^ These authors are equal contributions to this work.

^*^ Corresponding authors: DX, E-mail: xdp17730205951@163.com; HZ, E-mail: haishengs@ahmu.edu.cn

***Supplementary Table.***

**Table S 1.** List of Oligonucleotide Primers

| **Gene** | **Gene ID** | **Forward primer  (5'- to -3')** | **Reverse primer  (5'- to -3')** | **PCR products (bp)** |
| --- | --- | --- | --- | --- |
| *GAPDH* (human) | 2597 | TCACTGCCACTCAGAAGACTGT | CGTTCAGCTCTAGGATGACCTT | 135 |
| *TLR7*（human) | 51284 | TCCTTGGGGCTAGATGGTTTC | TCCACGATCACATGGTTCTTTG | 79 |
| *TNF-α* (human) | 7124 | TGCACTTTGGAGTGATCGGC | CTCAGCTTGAGGGTTTGCTAC | 146 |
| *IL-6* (human) | 3569 | GGTACATCCTCGACGGCATC | CACCAGGCAAGTCTCCTCAT | 167 |
| *IL-23* (human) | 51561 | CACTAGTGGGACACATGGATCT | AGAGAAGGCTCCCCTGTGAA | 199 |
| *Gapdh* (mouse) | 14433 | ACCCTTAAGAGGGATGCTGC | CCCAATACGGCCAAATCCGT | 130 |
| *Tnf-α* (mouse) | 21926 | CGCTCTTCTGTCTACTGAACTTCGG | GTGGTTTGTGAGTGTGAGGGTCTG | 113 |
| *Il-6* (mouse) | 16193 | CTTCTTGGGACTGATGCTGGTGAC | TCTGTTGGGAGTGGTATCCTCTGTG | 91 |
| *Il-23* (mouse) | 83430 | AATAATGTGCCCCGTATCCAGT | GCTCCCCTTTGAAGATGTCAG | 142 |

***Supplementary Figures***

***Supplementary Figure 1.***

***
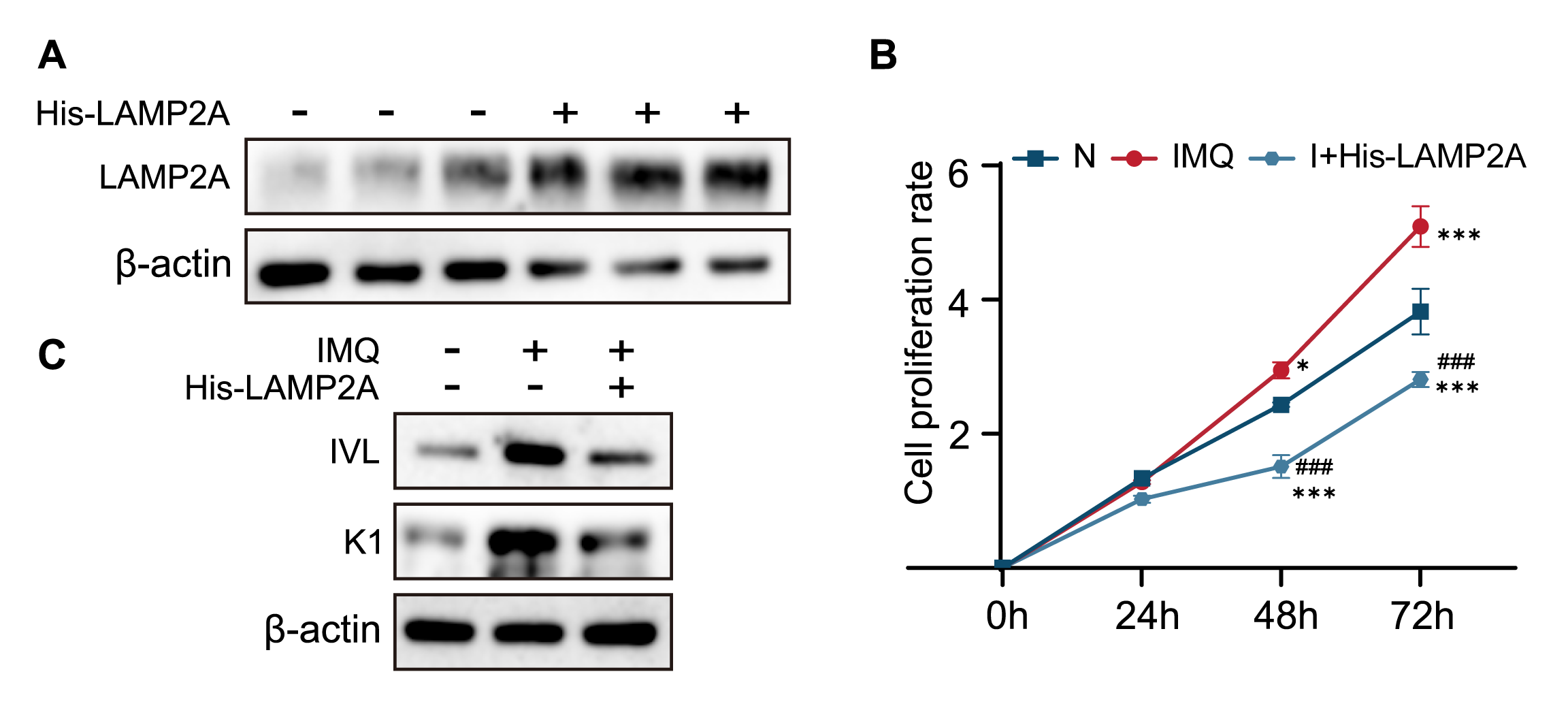
*Figure S1**. LAMP2A overexpression in response to IMQ-induced keratinocyte proliferation and differentiation. A. HaCaT cells were transiently transfected with pcDNA3.1-HisC-LAMP2A for 12 hours. LAMP2A overexpression was confirmed by immunoblotting. B. HaCaT cells were transiently transfected with pcDNA3.1-HisC-LAMP2A for 12 hours and then treated with IMQ (4.2 μM) for 24-72 h. Cell proliferation was assessed by CCK-8 assay. **P*<0.05, ***P*<0.01, and ****P*<0.001, *vs* the non-treated (N) group; ^###^*P*<0.001, *vs* the IMQ-treated group. C. HaCaT cells were transiently transfected with His-LAMP2A for 12 hours, and were then treated with IMQ (10.4 μM) for 72 hours, Involucrin (IVL) and cytokeratin 1 (K1) expression was detected by immunoblotting.

***Supplementary Figure 2.***

**
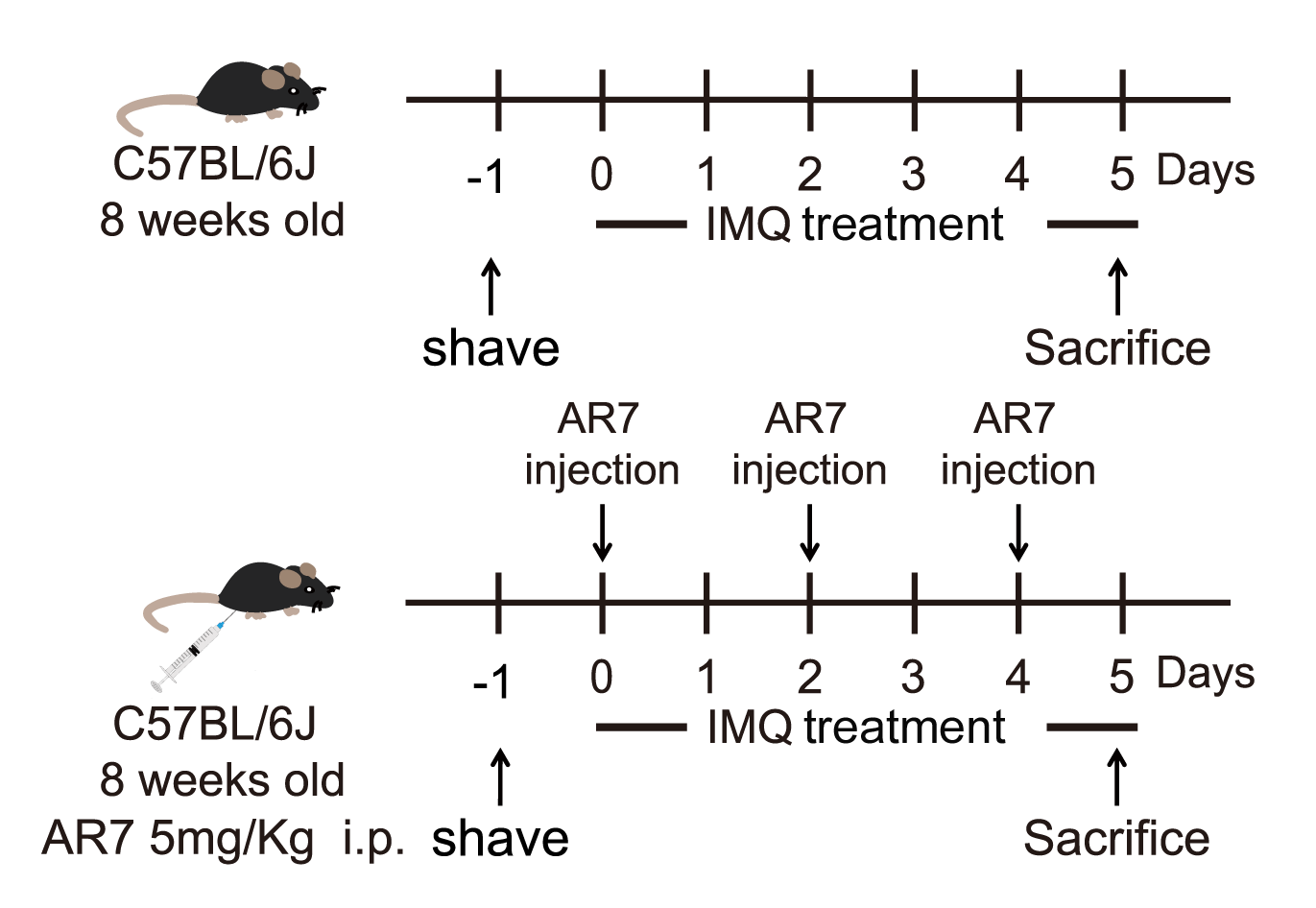
Figure S2**. Schematic diagram of the animal experimental procedure. Detailed procedures are described in the "Experimental Animals" section of the main text. IMQ: 5% imiquimod cream; AR7: atypical retinoid 7 (a CMA activator).

***
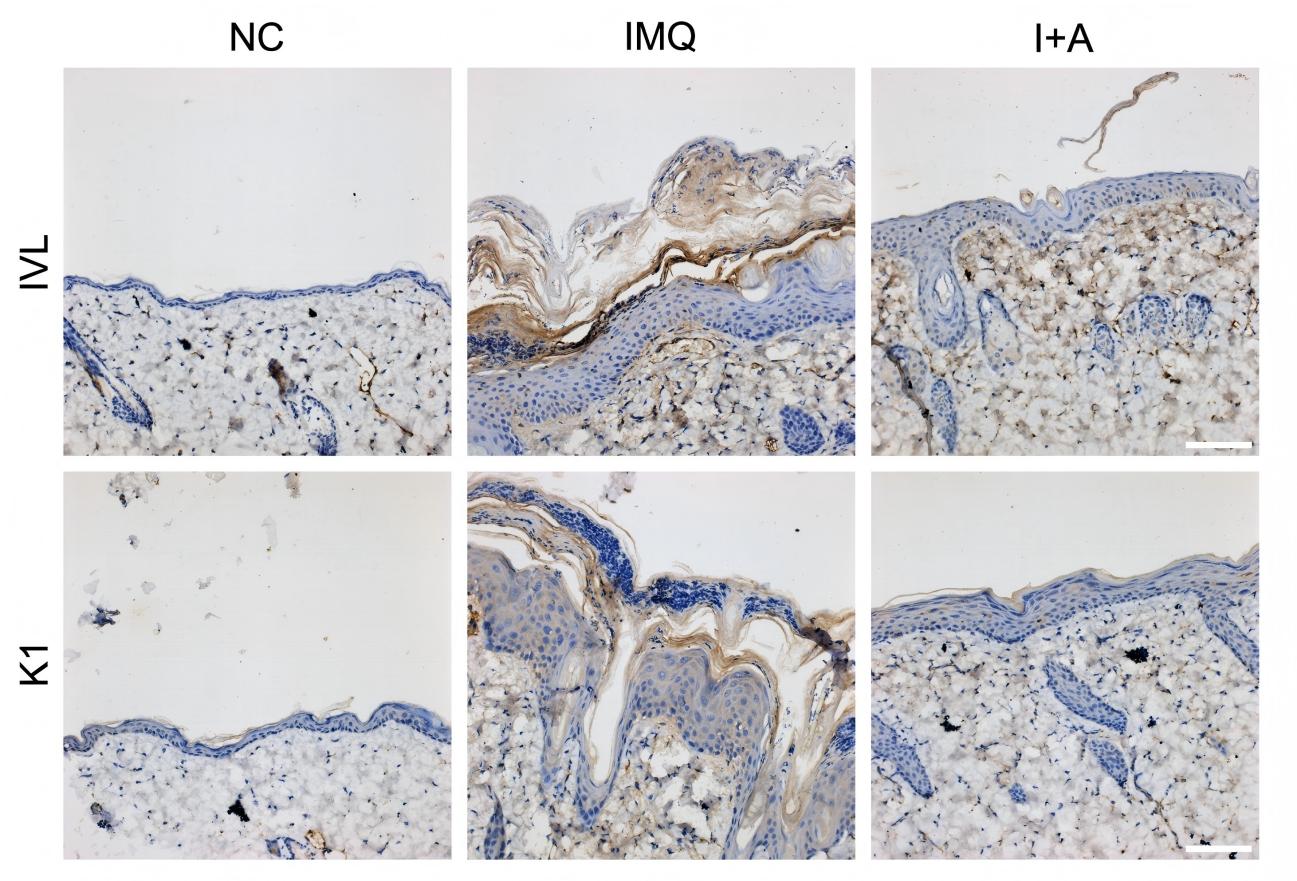
Supplementary Figure 3.***

**Figure S3**. Immunohistochemical Analysis of involucrin (IVL) and cytokeratin 1 (K1) in mouse dorsal skin. NC, normal skin; IMQ, imiquimod-induced psoriasiform lesions; I+A, IMQ-induced psoriasiform lesions with AR7 treatment. Scale bar=100 μm.

***Supplementary Figure 4.***

**
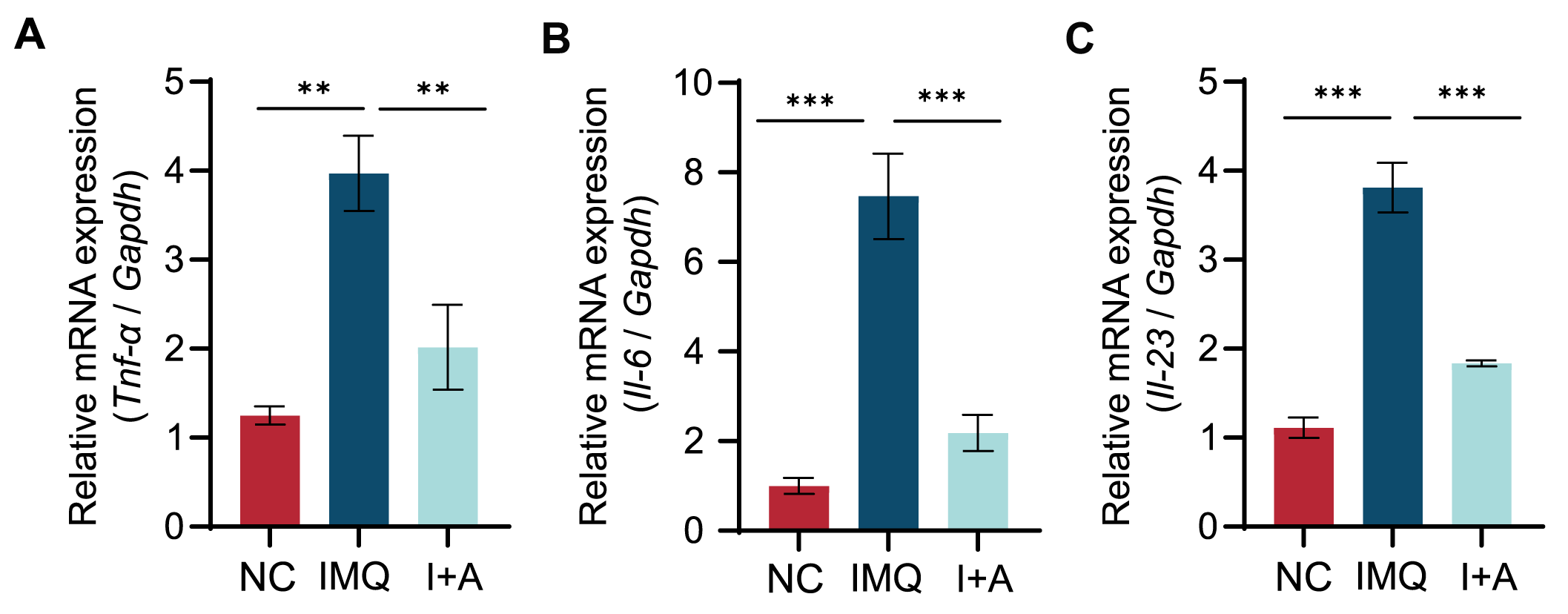
Figure S4**. Quantitative real-time PCR analysis of cytokines expression levels in mouse skin lysates (n=4 per group). A. Relative mRNA expression of *TNF-α; B.*Relative mRNA expression of *IL-6*; C. Relative mRNA expression of *IL-23*. ***P*<0.01, and ****P*<0.001. NC, normal skin; IMQ, imiquimod-induced psoriasiform lesions; I+A, IMQ-induced psoriasiform lesions with AR7 treatment.

***Supplementary Figure 5.***

***
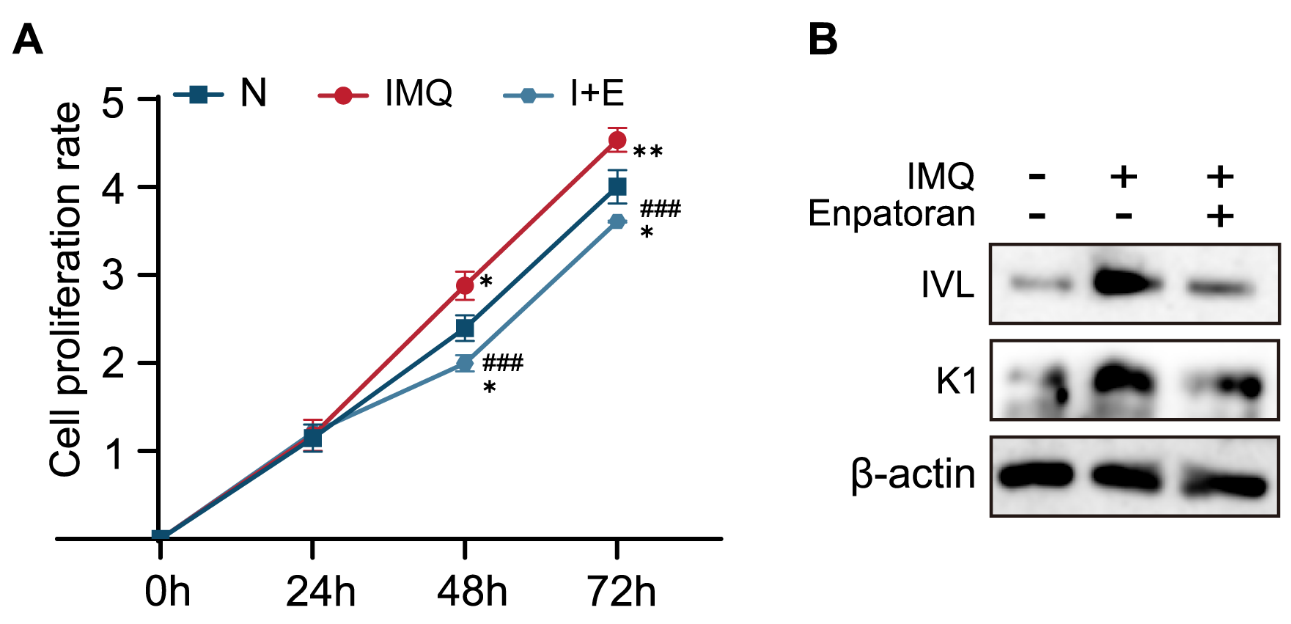
***

**Figure S5.** Effects of a TLR7 inhibitor on IMQ-induced responses in keratinocytes. A. HaCaT cell proliferation was assessed by CCK-8 assay after treatment with IMQ (4.2 μM) and/or enpatoran (5.0 μM) for 24-72 h. **P*<0.05, ***P*<0.01, and ****P*<0.001, *vs* the non-treated (N) group; ^###^*P*<0.001, *vs* the IMQ-treated group. B. Involucrin (IVL) and cytokeratin 1 (K1) expression in HaCaT cells was assessed by immunoblotting. Cells were cultured in KGM for 5 days, then treated with IMQ (10.4 μM) alone for 72 hours or pretreated with enpatoran (5.0 μM) for 48 hours followed by IMQ for 72 hours.

***Supplementary Figure 6.***

**
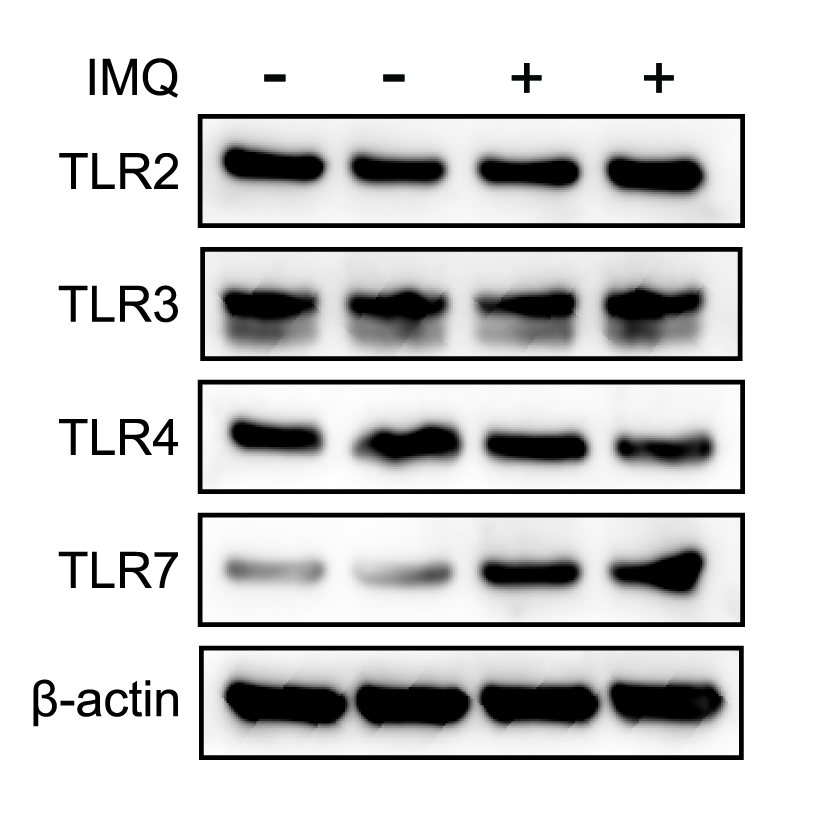
Figure S6.** TLR expression in HaCaT cells in response to IMQ. HaCaT cells were treated with IMQ (10.4 μM) for 3 days. Total protein lysates were then prepared and analyzed for TLR expression by immunoblotting.
